# Maternal hyperglycemia during labor and related immediate post-partum maternal and perinatal outcomes at the Yaoundé Central Hospital, Cameroon

**DOI:** 10.1186/s41043-016-0065-x

**Published:** 2016-08-22

**Authors:** Manuella Djomhou, Eugene Sobngwi, Jean Jacques N. Noubiap, Mickael Essouma, Philip Nana, Nelson J. Fomulu

**Affiliations:** 1 Department of Internal Medicine and Specialties, Faculty of Medicine and Biomedical Science, University of Yaoundé I, Yaoundé, Cameroon; 2 Laboratory for Molecular Medicine and Metabolism, Biotechnology Center, University of Yaoundé I, Yaoundé, Cameroon; 3 National Obesity Center, Yaoundé Central Hospital and Faculty of Medicine and Biomedical Sciences, University of Yaoundé 1, Yaoundé, Cameroon; 4 Department of Medicine, University of Cape Town and Groote Schuur Hospital, Cape Town, South Africa; 5 Medical Diagnostic Center, Yaoundé, Cameroon; 6 Department of Obstetrics and Gynecology, Faculty of Medicine and Biomedical Science, University of Yaoundé I, Yaoundé, Cameroon

**Keywords:** Maternal hyperglycemia, Gestational diabetes, Maternal and perinatal outcome, Immediate post-partum, Macrosomia, Sub-Saharan Africa

## Abstract

**Background:**

Data on the prevalence and complications of gestational diabetes are very scarce in Cameroon. The aim of this study was to evaluate the uptake of screening for gestational diabetes and assess the immediate post-partum outcome of hyperglycemic parturient mothers and perinatal outcome of their babies.

**Methods:**

A prospective cohort study was held at the Maternity of the Yaoundé Central Hospital from March to June 2013. One hundred volunteer women in labor without overt diabetes mellitus and having fasted for 8 to 12 h were recruited. No intervention was given. A clinical examination was done and capillary glucose recorded. Parturient women were categorized into two groups (hyperglycemic and non-hyperglycemic subjects) based on glycemia results interpreted according to the International Association of Diabetes and Pregnancy Study Groups. Mothers’ clinical examination was repeated and neonates examined immediately after delivery. Perinatal outcomes associated with maternal hyperglycemia during labor were assessed using relative risks. A *p* value <0.05 was considered statistically significant.

**Results:**

One hundred women with a mean age of 27 (SD 6) years were recruited. Of them, 22 (22 %) had already been screened for gestational diabetes at baseline. Thirty-one (31 %) were diagnosed with hyperglycemia during labor, and this condition was highly associated with macrosomia in neonates (RR = 8.9, 95 % CI 2.70–29.32; *p* < 0.001). Other complications associated with maternal hyperglycemia during labor were perineal tears, cesarean section, and intrauterine fetal death, though the association was not statistically significant.

**Conclusions:**

The main finding of this study is that maternal hyperglycemia during labor is highly associated with macrosomia in neonates. About a third of mothers were concerned with hyperglycemia during labor, and gestational diabetes was insufficiently screened in this series.

## Background

According to the World Health Organization (WHO), gestational diabetes mellitus (GDM) stands for any degree of glucose intolerance, with onset or first recognition during pregnancy, no matter the necessary treatment and post-partum evolution [1, 2]. It makes up to 90–95 % of cases of diabetes mellitus seen in pregnant women [3]. There are conflicting reports about its screening, adequate diagnostic tools, and adapted fasting glucose thresholds [4]. However, the most convincing study on adverse outcome associated with maternal glucose intolerance to date is the HAPO (Hyperglycemia and Adverse Pregnancy Outcomes) study [5, 6], from which most of internationally acceptable criteria for diagnosis and classification of GDM are derived. GDM prevalence ranges from 2 to 22 % of all pregnancies. Ethnicity, the screening method, and the glucose threshold used can explain this wide gap. Of note, prevalence increases with decreasing glucose thresholds. In the United States of America (USA), the prevalence of GDM was 2.8 % in 2007; it was 3.8 and 4.5 % in France in 2004 and 2005, respectively [7]. In sub-Saharan Africa, the prevalence of GDM varies from 3.7 % in Ethiopia (1999) to 3.8 % in South Africa (2007) and 6.3 % in Cameroon (2013) [8–10].

Previous studies report a number of maternal and fetal complications of GDM. However, their proof is limited by the lack of a consensus definition which makes their results difficult to compare. Furthermore, poor outcomes associated with different degrees of impaired glucose tolerance can be explained by a number of confounding characteristics such as obesity, an older maternal age, and maternal comorbid conditions which are not usually adjusted in those studies [6]. With respect to conflicting reports on screening and diagnosis of GDM, questions can be raised about cost-effectiveness and benefit of screening and treating GDM [6], especially in resource-limited countries such as Cameroon. So, it is not yet systematically screened, and a number of cases can therefore be missed, leading to a high risk of maternal and fetal adverse outcome. In order to assess the impact of missed opportunities of diagnosis, we carried out this study with an aim to evaluate the uptake of screening for GDM and assess the immediate post-partum outcome of hyperglycemic parturient mothers and perinatal outcome of their babies.

## Methods

### Setting, participants, and procedure

We conducted a prospective cohort study between March and June 2013 at the Maternity of the Yaoundé Central Hospital. At baseline, we included all pregnant women who came to deliver at the study site during the study period, who had fasted for 8 to 12 h, and who consented to participate in the study. We excluded women with overt diabetes mellitus. No intervention was given. Each participant underwent a thorough clinical evaluation, with focus on demographic parameters, pregnancy follow-up and evolution details, past medical comorbid conditions, and present physical examination’s findings. A capillary glycemia was performed, and women were divided into two groups (non-hyperglycemic and hyperglycemic individuals), based on glycemia results interpreted as recommended by the International Association of Diabetes and Pregnancy Study Groups (IADPSG) criteria. The HAPO study was used as the basis of these criteria [6]. Women were then followed up during the labor in delivery rooms until delivery. Immediately after delivery, they were re-examined. Newborns were also examined and followed up. All women and newborns were followed up for at least 48 h. In case a perinatal complication occurred, the follow-up period was prolonged until the health status of the woman or the newborn was stabilized or death occurred.

### Statistical analyses

This was a pilot study based on a convenient sample and, accordingly, no formal power estimation. Data were entered, coded, and analyzed using Epi-info 3.5.3 software. Variables are expressed as mean with standard deviation (SD) or count with percentage. The chi-square test or the Fischer exact test where appropriate were used to compare proportions. Relative risk was used to assess the perinatal outcomes associated with maternal hyperglycemia during labor. A *p* value <0.05 was considered statistically significant.

## Results

A total of 100 women were recruited. Their mean age was 27 (SD 6) years, with 60 % of them aged between 20 and 30 years. All the participants were educated. The high school level of education was the most represented (56 %, *n* = 56), followed by the university level (32 %, *n* = 32), and then the primary level (29 %, *n* = 29). Only 22 (22 %) of them had had an antenatal screening or diagnosis of gestational diabetes at baseline. And of these, only one (5 %) had been screened between the 24th and the 28th week (Table 1). Thirty-one parturient women (31 %) had hyperglycemia (Table 2).

**Table 1:** Antenatal screening/diagnosis of gestational diabetes in parturient women

Variables	Number	Percentage
Antenatal screening of gestational diabetes (*N* = 100)		
Yes	22	22
No	78	78
Gestational age (in weeks) at screening (*N* = 22)		
<24	17	77
[24–28]	1	5
>28	4	18

**Table 2:** Maternal glycemia during labor

Glycemia (g/l)	Number	Percentage
<0.80	40	40
0.80–0.91	29	29
≥0.92	31	31

As shown in Table 3, perineal tears, cesarean section delivery, intrauterine fetal death, and neonatal macrosomia were all related to maternal hyperglycemia. But the relation was significant only for neonatal macrosomia (RR = 8.9, 95 % CI 2.70–29.32; *p* < 0.001).

**Table 3:** Maternal and perinatal complications related to maternal hyperglycemia

Complications	Hyperglycemia (*N* = 31)	Non-hyperglycemia (*N* = 69)	Relative risk	95 % CI	*p* value
Maternal complications					
Perineal tears	4	6	1.48	0.45–4.89	0.52
Caesarian section	6	8	1.67	0.63–4.40	0.30
Instrumental delivery	2	0	10.93	0.54–221.33	0.11
Post-partum hemorrhage	1	0	6.56	0.27–156.74	0.24
Perinatal complications					
Macrosomia	12	3	8.90	2.70–29.32	<0.001
Intrauterine death	3	0	15.31	0.81–287.79	0.06
Premature delivery	4	8	1.11	0.36–3.42	0.85
Respiratory distress	2	0	10.93	0.54–221.33	0.11
Rapid neonatal death	0	1	0.72	0.03–17.41	0.84
Intrapartum death	0	1	0.72	0.03–17.41	0.84

*95 % CI* 95 % confidence interval

## Discussion

This study confirms the hypothesis of a very low rate of detection of GDM in our clinical settings, with only approximately a quarter of parturient women who were well previously screened for this condition. Meanwhile, about a third of delivering women were diagnosed with hyperglycemia in the labor room, and this condition was highly associated with neonatal macrosomia.

Most of the delivering women missed the opportunity of GDM detection early in the pregnancy. This reflects the non-application of available guidelines regarding detection and management of GDM in our clinical settings. In fact, it is recommended to realize GDM detection as from the first antenatal visit by a fasting glycemia and an oral tolerance glucose test at 24–28 weeks’ gestation, to prevent an adverse pregnancy outcome [6, 11]. A low rate of antenatal screening of this condition is a well-known problem. Notably, adequate policies of GDM screening were only recently drawn [11]. Also, many of the patients in this series had been followed up elsewhere for their pregnancy and were referred to the Yaoundé Central Hospital for complicated pregnancy termination. Even though most women had at least a high school level, they were not screened for GDM. Thus, the pregnant woman’s level of education might not influence the clinician’s practice concerning the detection and management of GDM.

However, the rate of maternal hyperglycemia was 31 %. This is much higher than the rate of GDM (5.2 %) found in Kinshasa in 2010 [12]. It is still much higher than the 5–17 % rate of GDM found in Cameroon of the same year (unpublished data). Those studies were carried out before the IADPSG criteria were published. They used different diagnostic criteria. It is therefore difficult to compare their results to those of this study. Furthermore, it is well known that using the HAPO thresholds increases the proportion of women diagnosed with GDM, since it proved that even at lesser maternal glucose thresholds than those diagnosed with diabetes, there is an increased risk of adverse maternal and perinatal outcome [6]. This proportion could have been more elevated if we had added an intervention such as the 1-h glucose test [6]. Maternal hyperglycemia and GDM have been associated with an adverse maternal and perinatal outcome, as we found in this study. We observed a non-significant association between perineal tears, cesarean section delivery, intrauterine fetal death, and maternal hyperglycemia. These are well-known complications found in pregnant women presenting with impaired glucose tolerance. But the effect of hyperglycemia in generating these troubles seems minor, and they are usually attributed to cofounders such as obesity, an older maternal age, and medical comorbid conditions co-occurring in GDM patients, rather than to hyperglycemia per se [6, 11]. Maternal impaired glucose tolerance also prompted a high risk of birth weight large for gestational age (LGA) in this study, same as Odar et al. in Uganda in 2004 [13]. The risk of LGA found in this study was even approximately double of that found in their study. The HAPO as well as most of all interventional and cohort studies evaluating the perinatal outcome of maternal hyperglycemia report an increased risk of LGA. The risk of LGA has a continuous graded relationship with higher maternal glucose levels [6]. This can be detected even at lesser glucose levels such as 0.92 g/l used in this study [6, 11]. A higher glucose threshold in the study of Odar et al. could thus have decreased the risk they observed. LGA usually occurs with excess neonate’s adiposity, neonatal hypoglycemia, and excess neonate’s cord C-peptide [6]. In fact, excess maternal blood glucose is transported to the fetal circulation through the placenta. This is firstly responsible for increase of blood glucose which excessively stimulates insulin production. Fetal hyperinsulinemia then induces hypoglycemia observed in the neonate through its anabolic and glycogenolytic pathways. Anabolic reactions are also responsible for excess adiposity and macrosomia [6]. And LGA can lead to overweight/obesity and even diabetes mellitus in the mother as well as in the infant [6, 11].

This study has some limitations. The follow-up of parturient women makes it possible to draw causal relationships between maternal hyperglycemia and immediate maternal and perinatal complications in our setting. But the short duration of follow-up and the lack of some neonatal biological analyses such as calcemia, bilirubinemia, and full blood count have limited the information that could have been brought by this study. Also, we did not adjust for confounders. However, the susceptibility to LGA in case of maternal glucose intolerance that we observed is said to be independent of confounders. This might therefore not have affected our finding. Lastly, there is not a direct or causal relationship between maternal hyperglycemia during labor and neonatal macrosomia, as maternal hyperglycemia during labor is only an indicator of probable maternal hyperglycemia during the third trimester of pregnancy which is the actual correlate of neonatal macrosomia.

## Conclusions

Maternal hyperglycemia during labor was highly associated with neonatal macrosomia. Maternal hyperglycemia was present in about a third of mothers, and antenatal screening of gestational diabetes was poor in this setting. Future large longitudinal prospective and interventional studies are warranted to confirm these findings.

## Abbreviations

WHO: World Health Organization
GDM: Gestational diabetes mellitus
IADPSG: International Association of Diabetes and Pregnancy Study Groups
HAPO: Hyperglycemia and Adverse Pregnancy Outcomes
LGA: Birth weight large for gestational age

## References

[1] World Health Organization (2006). The global burden of disease 2000.

[2] IDF-Africa, WHO-AFRO (2006). The diabetes strategy for Africa. An integrated strategic plan for diabetes and related health risk.

[3] Petitti DB, DeWitt TG, Gordis L, Gregory KD, Harris R, Isham G (2008). Screening for gestational diabetes mellitus: U.S. Preventive Services Task Force recommendation statement. U.S. Preventive Task Force. Ann Intern Med.

[4] Haute Autorité de Santé (2005). Rapport de synthèse sur le dépistage et le diagnostic du diabète gestationnel.

[5] Metzger BE, Lowe LP, Dyer AR, Trimble ER, Chaovarindr U, HAPO Study Cooperative Research Group (2008). Hyperglycemia and adverse pregnancy outcomes. N Engl J Med.

[6] International Association of Diabetes and Pregnancy Study Groups Consensus panel (2010). International Association of Diabetes and Pregnancy Study Groups recommendation on the diagnosis and classification of hyperglycaemia in pregnancy. Diabetes Care.

[7] Venditteli F, Rivière O, Crenn-Hébert C, Claris O, Tessier V, Pinquier D (2008). Audipog perinatal network. Part 1: principal perinatal health indicators, 2004–2005. Gynecol Obstet Fertil.

[8] Mamabolo RL, Alberts M, Steyn NP, Delemarre-van dewaal HA, Levitt NS (2007). Prevalence of gestational diabetes mellitus and the effect of weight on measures of insulin secretion and insulin resistance in third-trimester pregnant rural women residing in the central region of Limpopo province, South Africa. Diabet Med.

[9] Seyoum B, Kiros K, Haileselase T, Leole A (1999). Prevalence of gestational diabetes mellitus in rural pregnant mothers in northern Ethiopia. Diabetes Res Clin Pract.

[10] Jao J, Wong M, Van Dyke RB (2013). Gestational diabetes mellitus in HIV-infected and -uninfected pregnant women in Cameroon. Diabetes Care.

[11] World Health Organization (2013). Diagnostic criteria and classification of hyperglycemia first detected in pregnancy.

[12] Tandu-Umba NFB, Paka MA, Mbungu MR, Muls E (2010). Détermination de la prévalence du diabète gestationnel et des facteurs associés à Kinshasa. Ann Afr Med.

[13] Odar E, Wandabwa J, Kiondo P (2004). Maternal and fetal outcome of gestational diabetes mellitus in Mulago Hospital, Uganda. Afr Health Sci.

